# Microbial and Quality Changes of Seabream Fillets Processed with Cold Plasma During Refrigerated Storage

**DOI:** 10.3390/foods14091443

**Published:** 2025-04-22

**Authors:** Silvia Tappi, Lorenzo Nissen, Ana Cristina De Aguiar Saldanha Pinheiro, Fabio D’Elia, Flavia Casciano, Giorgia Antonelli, Elena Chiarello, Francesca Soglia, Giulia Baldi, Filippo Capelli, Andrea Gianotti, Alessandra Bordoni, Massimiliano Petracci, Francesco Capozzi, Marco Dalla Rosa, Pietro Rocculi

**Affiliations:** 1 Department of Agricultural and Food Sciences (DISTAL), University of Bologna, Piazza Goidanich 60, 47521 Cesena, Italy; silvia.tappi2@unibo.it (S.T.); lorenzo.nissen@unibo.it (L.N.); f.delia@food-hub.it (F.D.); cascianoflavia@yahoo.it (F.C.); giorgia.antonelli4@unibo.it (G.A.); elena.chiarello2@unibo.it (E.C.); francesca.soglia2@unibo.it (F.S.); giulia.baldi24@gmail.com (G.B.); andrea.gianotti@unibo.it (A.G.); alessandra.bordoni@unibo.it (A.B.); m.petracci@unibo.it (M.P.); francesco.capozzi@unibo.it (F.C.); marco.dallarosa@unibo.it (M.D.R.); pietro.rocculi3@unibo.it (P.R.); 2 Interdepartmental Centre for Industrial Agri-Food Research (CIRI), University of Bologna, Piazza Goidanich 60, 47521 Cesena, Italy; 3 Department of Industrial Engineering (DIN), Alma Mater Studiorum-Università di Bologna, Via Terracini 24, 40131 Bologna, Italy; filippo.capelli@unibo.it; 4 Almaplasma s.r.l., Viale Giuseppe Fanin, 48, 40127 Bologna, Italy

**Keywords:** shelf life, non-thermal processing, lipid oxidation, protein oxidation, sensory acceptability

## Abstract

Cold plasma (CP) is a non-thermal technology, successfully used to decontaminate and extend the shelf-life of various foods. However, CP can cause quality deterioration in sensitive matrices, such as fish products. This research aimed to evaluate the effect of CP treatment obtained using different gas mixtures (80% Ar/20% O_2_, or 80% N_2_/20% O_2_) with a surface dielectric barrier discharge (SDBD) on the decontamination of spoilage microflora, the main quality indices and the sensory acceptability of seabream (*Spaurus aurata* L.) fillets during refrigerated storage. At the beginning and at the end of the shelf life, lipid and protein oxidation indices and the fatty acid profile were evaluated. Results showed that, despite a low initial microbial decontamination (0.2–0.3 Log CFU/g), an inhibition of the growth of the main spoilage bacteria was observed resulting in an increase of the microbiological shelf life of around 40% for both treatments. Although a slight increase in lipid and protein oxidation was observed (up to around 5 mg MDA/kg and 4 nmol/mg of protein for TBARs and carbonyl content respectively), the sensory acceptability was higher for plasma treated samples, while the fatty acid profile was not affected and only a slight variation in the surface colour was observed (L* value increase by 3 points), confirming that CP could represent an interesting strategy to extend the shelf life of seafood products with minimal impact on quality and nutritional value.

## 1. Introduction

Seafood and seafood products are highly perishable foods due to their chemical composition, high moisture content and polyunsaturated fatty acids. Fish fillets are characterized by a short shelf life, therefore strategies to improve their duration could be very appreciated by the industry. Various processing methods are used to extend shelf-life while maintaining organoleptic quality and minimizing loss of nutritional value. Recently, emerging non-thermal technologies have demonstrated their effectiveness in inactivating microorganisms and enzymes without compromising the sensory and nutritional quality [1]. In fact, if not properly optimized, traditional thermal processing can promote heat-induced changes in flavor and texture, due to oxidation and loss of juice. Non-thermal technologies may represent an advancement in seafood preservation, as also due to consumers trend to pay attention to the “clean label”, which involves the elimination of additives and promotes environmentally friendly processing techniques that require less energy and water [2].

Cold plasma (CP) is a new low-cost technology that is considered environmentally friendly because it requires no solvents and the energy demand is low [3]. CP is obtained by ionization of a gas mixture in which several chemical processes (ionization, excitation, dissociation etc.) are set in motion, resulting in large quantities of non-equilibrium species, among which several short- and long-lived species (atoms, molecules and radicals in grounded and excited forms, electrons, positive and negative ions, free radicals, gas atoms and radiations) are present. The effects on food preservation (inactivation of microorganisms, inhibition of enzymes activity, and structural changes) are based on different mechanisms related to the production of different reactive species depending on the gas mixture (O_2_, N_2_, CO_2_, Ar) and the type of generators used.

The use of CP has been studied in numerous food products, including fruits and vegetables, fresh meat and ready-to-eat meat products, and food packaging materials [4]. Regarding fish products, various reports showed its potential for microbial decontamination and shelf-life extension of fresh [5,6,7,8,9] and dry products [10,11,12,13,14]. However, in seafood products, the application of CP may induce the development of oxidative reactions affecting the lipid fraction leading to the formation of volatile compounds causing undesirable flavors [15]. Results are often conflicting, indicating a complex interaction between plasma reactive species and fish lipids [16].

In a previous work [17], upon CP exposure we observed a reduction in the microbial load of fresh seabream fillets, with little changes in product quality and nutritional value. Therefore, the aim of the present study was to evaluate whether CP treatment can extend the shelf-life of refrigerated seabream fillets. For this purpose, the most promising treatments selected in the previous study (Plasma-Air-20 min and Plasma-Argon-20 min) were applied to seabream fillets before packaging and storage. The main spoilage microorganisms and quality indices were evaluated over a two-week period of refrigerated storage and compared with those observed in the untreated sample.

## 2. Materials and Methods

### 2.1. Raw Material Preparation

Seabream (*Spaurus aurata* L.) (provided by Galaxidi, Greece), of an average weight of around 250 g, were received at the company EMAR (Economia del Mare, Cesenatico, Italy) facilities, where they were gutted, skinned and filleted and subjected to fast freezing at −45 °C. Frozen samples were stored at −45 °C for 4 weeks before experiments. The freezing of samples was needed for practical reasons, in order to plan and execute the experiments. At −45 °C lipid oxidation is supposed to be significantly slowed down, as reported by Passi et al. [18], who evidenced that deep-freezing of fish muscle at −30 °C and −80 °C for up to 12 months did not significantly affect the levels of enzymatic antioxidants, and of polyunsaturated fatty acids. Before CP treatment, fillets were thawed overnight at 2 ± 1 °C. Each treatment was carried out on at least 6 fillets at the same time. The average weight of fillets was 53 ± 6.5 g.

### 2.2. Plasma Treatment

The prototype device used in this research was described in Capelli et al. [19] and was composed of a generator, a plasma source and a treatment chamber. The plasma source was a Surface Barrier Discharge (SBD), powered by a high voltage pulse generator (mod. S-P300, Alintel S.r.l., Bologna, Italy). The climatic chamber allowed to control treatment time and gas mixture (different combinations of O_2_, N_2_, CO_2_, Ar, N_2_O) through the connection to a quaternary gas mixer (mod. KM100-4, Witt-Gasetechnik, Witten, Germany).

The selected processing parameters were 5 kHz, 18 kV, 20 min, and the selected gas mixtures were 80% N_2_/20% O_2_ (Plasma-Air) and 80% Ar/20% O_2_ (Plasma-Argon). As reported in Tappi et al. [17], after 20 min the ozone concentration reached 4091 ± 237 ppm for plasma-argon and 5018 ± 312 ppm for plasma-air. NO_2_ concentration was not detectable, and the temperature within the chamber was 29 °C for air and 32 °C for argon. During the treatment, a core temperature between 5 (start)–14 °C (end) was measured in the fillets.

### 2.3. Packaging and Storage

After the CP treatment, the fillets were packed using modified atmosphere packaging (20% CO_2_–80% N_2_) obtained using a quaternary gas mixer (mod. KM100-4, Witt-Gasetechnik, Witten, Germany). Product volume to gas ratio was about 1:1. Each fillet was individually packed in polypropylene (PP) trays sealed with high barrier PP film using a packaging machine (mod. VGP, ORVED, Venezia, Italia), and stored at 3 ± 1 °C. Analytical determinations were carried out after 0, 2, 6, 9 and 13 days of refrigerated storage on at least 8 individually packaged fillets for each treatment and sampling time.

### 2.4. Analytical Determinations

#### 2.4.1. Microbiological Analysis

Microbiological analysis, as the plate count technique was done to enumerate live microbial cells. The methods followed were ISO 4833-1:2013 [20], ISO 21528-2:2017 [21], and ISO 17410:2019 [22], for mesophiles, Enterobacteriaceae and psychrophiles respectively, with little modifications. Briefly at each time points, ten grams of flesh were recovered from three fillet replicates and two technical replicates were prepared by independent homogenization with 90 mL of saline solution (0.9 g/L NaCl) using sterile filtered bags (Interscience, Cantal, France) and a stomacher apparatus at 120 paddle/min for 2 min (Seward, Worthing, UK). Serial dilutions of the homogenates were then prepared with sterile saline solution in glass tubes. Selected dilutions were then plated on sterile agar-based media (Oxoid, Thermo Fisher, Walhtam, MA, USA) and incubated at specific conditions, namely: on Plate Count Agar (PCA) at 30 °C for 48 h for mesophiles; on Violet Red Bile Glucose Agar (VRBGA) at 37 °C for 24–48 h for Enterobacteriaceae; on PCA at 5 °C for 7–10 days for psychrophiles. Enumeration of microbial load was calculated as a mean of three biological replicas (*n* = 3) and presented as Log CFU/g (Colony Forming Unit/g) ± Standard Deviation.

#### 2.4.2. Physico-Chemical Parameters

The pH was determined on of 5 g fish flesh homogenized in 5 g distilled water using a pH-meter (Crison, Barcellona, Spain). The color parameters lightness (L*), redness (a*) and yellowness (b*) were determined using a spectrophotocolorimeter mod. ColorFlex™ (Hunterlab, Reston, VA, USA) (CIE, 1976). Color as measured on surface of the whole fillet Dry matter was determined gravimetrically by drying in an oven at 70 °C to constant weight.

Texture was measured with a Texture Analyser mod. TA.HDi 500 (Stable Micro Systems, Godalming, UK) equipped with a 5 kg load cell. The fillet was subjected to a compression in its middle part with a cylindrical probe with a flat head, setting a descent rate of 3 mm/s with a total compression of 60%. The stress applied to the sample was measured through a stress-strain curve and the maximum force expressed in Newton (corresponding to the maximum peak reached during the compression of the sample) normalized by the sample weight (N/g) was considered and defined as ‘Hardness’.

Physico-chemical parameters were measured on five fillets.

#### 2.4.3. Sensory Evaluation

Sensory evaluation was carried out according to a modified quality index method (QIM) described in detail by [23]. The attributes examined were: (1) the development of slime on the surface of the fillet; (2) muscle incision and firmness; (3) odor; and (4) overall appearance of the fish fillet. Each assessment was carried out by a minimum of six trained panelists with a long-term training and experience in fish evaluation. Four categories were ranked: highest quality or excellent (E), good quality (A), fair quality (B), and unacceptable quality (C). Samples were coded with alphanumeric random codes, each of the panelists evaluated the same 3 fillets.

#### 2.4.4. Oxidative Indices

At the beginning of the storage and at the end of the microbiological shelf life, parameters describing the occurrence of lipid and protein oxidation were measured. In detail, ThioBarbituric Acid-Reactive Substances (TBARS) were quantified according to Bao and Ertbjerg [24] as index of lipid oxidation and expressed as mg of malonaldehyde (MDA) per kg of flesh. Protein oxidation was evaluated according to the method described by Soglia et al. [25] based on a spectrophotometric detection of the protein-bound hydrazones after their derivatization with 2,4-dinitrophenylhydrazine and expressed as nmol/mg of protein.

#### 2.4.5. Fatty Acids Profile

To evaluate the effect of the processing on the nutritional quality, the fatty acid (FA) profile and content (as methyl esters) was determined at the beginning of the storage and at the end of the microbiological shelf-life.

Total lipids were extracted from seabream fillets according to Bligh and Dyer [26] and the methylation of FA was performed adding 500 μL of hydrogen chloride solution 0.5 M in methanol (Sigma-Aldrich, Saint Louis, USA, 07607) at 100 °C for 1 h. At the end of the methylation step, 2 mL of hexane and 2 mL of distilled water were sequentially added. The hexane layer was transferred in a test tube and dried under nitrogen infusion. The resulting fatty acids methyl-esters (FAMEs) were suspended in 100 μL of hexane. The analysis of FAMEs was performed by fast-GC (GC-2030, Shimadzu, Kyoto, Japan) using a capillary column (30 mt, 0.2 μm film thickness) with a programmed temperature gradient (50–250 °C, 10 °C/min). The peaks were identified based on their retention time, which was predetermined using a standard mix solution (Supelco, Merck; Darmstadt, Germany, CRM47885), and quantified using Lab Solution Software, LCGC (Shimadzu, Japan) [27].

### 2.5. Statistical Analysis

Statistical analysis was carried out by the one-way ANOVA using Tukey’s-HSD as post-hoc test and assuming *p* < 0.05 as significant, with the software Statistica version 11.0 (Tibco Inc. Palo Alto, CA, USA).

## 3. Results and Discussion

### 3.1. Microbial Inactivation

The microbial decontamination efficiency of CP on fish products is generally variable and depends on many factors, such as the operating parameters, exposure mode (direct or indirect), gas mixture used and treatment time, that in turn, can influence the concentration of short and long-lived reactive species. In our previous research [17], we observed a lower inhibitory effect of the applied treatment in comparison with the results available in literature, highlighting however, a different sensitivity of inoculated Gram-positive and Gram-negative bacteria.

The results of the microbial load observed in the present study in the seabream fillets after CP treatment compared to the control are reported in Table 1. Initial levels of mesophiles were similar in all samples and in line with literature reference [7]. As previously observed [17], mesophiles appeared more sensitive to the Argon mixture than to the Air mixture. However, despite similar initial microbial loads their initial reduction was almost negligible (maximum 0.2 Log CFU/g of reduction for the plasma-Argon treatment), similarly to previous results (0.7 Log CFU/g of reduction for the Plasma-Argon treatment).

Compared to the control, a reduction of 0.3 Log CFU/g for *Enterobacteriaceae* and no significant differences for psychrophiles were observed in the Plasma-Air sample after treatment. This initial microbial reduction is modest, but triggers a constant microbial reduction during the whole shelf-life, whose trend is more intense than the trend of the control. In fact, despite the slight inhibition of bacterial load observed just after treatment, along the storage, a delay or inhibition in the growth of spoilage bacteria was observed, especially for *Enterobacteriaceae* and psychrophiles. Similar results were observed for mesophile growth in herring fillets treated with a Dielectric Barrier Discharge (DBD) in package plasma [5] and in seabass fillets washed with Plasma Activated Water [28]. The delayed growth can be explained due a bacteriostatic effect, however, to better discriminate between bacteriostatic and bactericidal effect, some further analysis such as flow cytometry or microscopic observation should be carried out. Moreover, the delayed growth could be due also to residual reactive species, as it has been reported that residual reactive species in packaged cold plasma-treated foods are active during the whole shelf-life [7].

According to standard references [29,30], the end of the shelf-life was considered as the time necessary to reach the microbiological load of 6 Log CFU/g for mesophiles and 4 Log CFU/g for *Enterobacteriaceae*. Mesophile load threshold was reached after 9 days for all the CP treated samples except for the control, but the Plasma-Air treatment seemed to slightly delay the microbial growth after this period. Similar outputs were previously observed by Giannoglou et al. [7].

For *Enterobacteriaceae*, considered as hygiene indicator, the latest time point of acceptability was between day 2 and day 6 for the control and at day 6 for Plasma-Air treated sample. Of note, the Plasma-Argon treated fillets did not reach the threshold during the 13-day study duration. It is conceivable that the greater effectiveness of the Argon mixture compared to that of Air is linked to the time necessary to reach the maximum ozone concentration in the plasma atmosphere. In fact, in the Argon regime the maximum concentration was reached after 10 min, while for the Air regime the maximum concentration was reached later on (20 min).

Lastly, the psychrophiles, which include those food-spoilage bacteria whose optimum temperature of growth is around 5 °C, had an initial load of around 3 Log CFU/g in all the samples. Starting from day 6, both plasma treated samples showed a load approximately 1 Log CFU/g lower than the control, and on day 13 the efficacy of treatments was similar. Control samples appeared not safe already after 6 days, while treated samples reached the same microbial load after 13 days of refrigerated storage, resulting in 7 days of longer microbiological safety, in comparison to the control.

Considering all microbial groups, treated samples were still safe after 9 days (and even further for Plasma-Argon) while controls were already unsafe between day 4 and 6 of storage. Therefore, it can be concluded that around a 40% increase in shelf life was attributable to plasma treatment.

As reported by Giannoglou et al. [7], among different non thermal technologies, there are differences in the effect on microbial inhibition. High Pressure processing shows high effectiveness, but causes a significant modification of color and texture of the fillets. On the other side, ozonation and pulsed electromagnetic fields appeared to be less effective in reducing the microbial load, compared to both HP and CAP processes.

### 3.2. Quality Parameters

The physico-chemical parameters measured in seabream fillets during storage are reported in Table 2. Neither the treatment nor the storage affected the dry matter content, which remained in the range 71–74%. Similarly, no differences were observed in the pH values. As for the color, no differences were observed for the a* value, while the b* value was slightly decreased after treatment and not during storage. The L* value increased in treated samples compared to controls. The changes were slight, although the observed difference (about 3 points) is considered the limit for detection by the human eye, and were evident until the 9th day of storage.

During storage, L* followed a different trend for the three samples, the control sample showed a slight but significant increase, that might be related to a protein denaturation effect. However, while some authors [31] reported a higher L* value in plasma treated samples during storage, generally a lower color variation is observed compared to the control, as reported by Chanioti et al. [32]. As the storage proceeded, a*-value gradually increased for all the samples (*p* < 0.05), possibly owing to the oxidation of haemoglobin, myoglobin, as well as heme-proteins. The modifications in color of fish fillets are widely reported as a consequence of plasma exposure, and are generally attributed to the oxidation of lipid and pigments and to protein denaturation [33]. However, results are often contrasting due to the complexity of the plasma-fish interactions. Indeed, while some authors observed a decrease in the L* values in herring and mackerel [5,6], an increase was reported by others [7,8,17].

**Figure 1:**
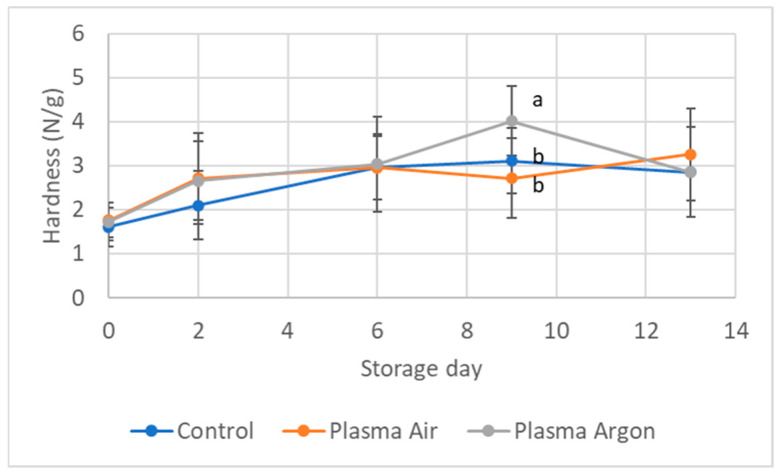
Texture parameter of Hardness (N/g) measured in the seabream fillets during refrigerated storage. Data are expressed as Log CFU/g sample and are mean ± SD of 3 biological replicates in each condition. Statistical analysis was by the one-way ANOVA using Tukeys’ as post-test and assuming *p* < 0.05 as significant. Different letters indicate statistical significance.

Figure 1 shows the texture of CP treated seabream fillets compared to controls during refrigerated storage. After CP treatment, no differences were detected in texture, although a slight increase of compression force was observed for all samples during storage. Similarly, other authors did not find any difference in textural parameters of fish fillets treated with CP, measured by instrumental tests [7] or by sensory analysis [8,9].

At T0, sensory analysis indicated that the quality of both control and treated seabream fillets was excellent (Table 3). Although sensory quality decreased during storage, on day 13 plasma-treated samples were still considered acceptable, while control samples reached the unacceptability score.

The oxidation level of the lipids (TBARS) and proteins (carbonyls) were assessed immediately after treatment and at the end of microbial shelf life (that was considered at day 9), and are reported in Table 4. TBARS level is a good indicator of fish quality, and is widely used to quantify the amount of secondary products of lipid oxidation [34]. The limit of acceptability correlated to the TBARS value is not clear and different authors report different threshold values, probably because it is strictly related to the type of fish. In fish stored with ice, it has been suggested that a TBARS value < 5 mg MDA/kg indicates good quality, while the fish may be consumed up to a level of 8 mg MDA/kg [35].

Immediately after CP treatment, TBARS level was not significantly affected; however, exposure to plasma reactive species triggered the lipid oxidation during storage, resulting in a final value of about 5 mg MDA/kg in the treated samples, compared to 1.6 mg MDA/kg of the control one. As the sensory quality of CP-treated fillets was considered fair until day 9, while only acceptable in the controls, we speculate that the increase of lipid oxidation was not determinant for acceptability, which was probably more correlated to the development of psychrophile microorganisms. Similar results were observed by Giannoglou et al. [7], who suggested they that the sensory profile of the treated fillets was ascribable to a reduced intensity of the perceived odor, in turn related to the decrease of volatile organic compounds (VOCs) that can be attributed to plasma-induced decomposition. Among these, trimethylamine is the main responsible for the fish off-odor. A determination of both volatiles and trimethylamine would therefore help to clarify this situation.

Protein oxidation negatively impacts on fish quality, inclusive of flavor, color, odor, and texture attributes [33]. Although no significant differences in protein oxidation between samples were detected at day 0, at the end of the shelf life both CP-treated fillets showed remarkably higher carbonylation levels than controls. This could be attributed to the modifications of certain amino acid side chain groups, in particular with NH− or NH_2_ or by peptide bond cleavages [36]. However, protein oxidation might impact also some functional properties, as texture and digestibility. While texture (Figure 1) does not show modifications that can be attributed to protein oxidation results, digestibility has not been measured in the present study.

Overall, these findings are in agreement with those available in the literature depicting a potential role of CP in promoting and accelerating the occurrence of oxidative phenomena affecting both the lipid and the protein fraction of aquatic food products [33,37,38,39,40]. As possible mitigation strategy, the use of antioxidant compounds (e.g., rosemary extracts) has shown some promising results for the prevention of fish lipids oxidation [41], in particular if combined with vacuum packaging [42].

### 3.3. Fatty Acids Composition

Since CP is an advanced oxidation technique and unsaturated lipids are sensitive to oxidation, many studies have focused on assessing the extent of lipid oxidation while relatively few studies have assessed changes in fatty acid composition. In wheat flour, Bahrami et al. [43] observed a reduction of fatty acids and phospholipid contents after CP treatment. Plasma treatment decreased the relative amount of unsaturated fatty acids even in dairy and meat samples [44].

In the present study, CP treatment carried out with both gas mixtures did not cause any significant modification in the fatty acid profile of fillets compared to controls either at day 0 or 9 (Table 5). It is acknowledged that the effect of CP on lipids is dependent on treatment time and power, and optimizing system parameters as well as storing at appropriate conditions can significantly reduce them [45]. It is conceivable that also the type of food matrix can modulate the effects of CP on the lipid profile. Indeed, similar results to those reported here were obtained by Pérez-Andrés et al. [39] in mackerel and by Kulawik et al. [38] in sushi.

Although variability in total fatty acid content (mg FAME/100 g sample) was detected among samples of the same group, expressing the content of individual fatty acids as a percentage of total fatty acid content (mol/100 mol), the variability appeared low. Nutritional indices (i.e., % SFA, MUFA, PUFA, n-3 and n-6 PUFA and n-6/n-3 ratio) had a low coefficient of variation and were similar in all conditions, except % n-6 PUFA in Plasma-Air vs Plasma-Argon treated fillets after 9 days of storage. The n-6/n-3 ratio, which should not be higher than 4 according to the nutritional recommendations [39], was similar in all conditions and in the range 0.5–0.6, indicating a good nutritional quality.

## 4. Conclusions

In the present study, the application of CP to seabream fillets allowed the extension of their microbial shelf-life. This was particularly evident using the Plasma-Argon mixture, which increased the shelf -life related to some microbial safety indicators by 40% compared to the untreated samples, which can be considered significant in such a perishable food. Although at the end of storage time a slight increase of oxidative indices was observed in CP treated fillets, it did not negatively influence the sensory perception. In addition, other qualitative indices and the fatty acids profile were not or only slightly affected, confirming that CP could represent an interesting strategy to extend the shelf life of seafood products with minimal impact on quality and nutritional value. However, there are few issues to consider in order to promote the industrial adoption of this technology. First, although cold plasma is considered a low-energy technology, and allows to avoid the use of chemical sanitizers, in order to understand the environmental impact and cost effectiveness, full life cycle and life cost assessments (LCA and LCC) should be carried out. Moreover, regulatory aspects should be carefully considered, as the EU Novel Food regulation might apply, and finally, the consumer perception related to this new technology should eb carefully considered.

## Figures and Tables

**Table 1:** Microbial loads (Log CFU/g ± SD from Log) of Mesophiles, *Enterobacteriaceae* and Psychrophiles in the seabream fillets during refrigerated storage.

Storage Day	Control	Plasma-Air	Plasma-Argon	*p* Value Treatment
Mesophiles				
0	3.08 ± 0.03 ^C^	3.05 ± 0.03 ^B^	2.81 ± 0.05 ^C^	0.066001
2	3.11 ± 0.04 ^C^	3.09 ± 0.06 ^B^	3.06 ± 0.04 ^C^	0.422850
6	3.88 ± 0.01 ^C^	3.91 ± 0.02 ^B^	3.73 ± 0.03 ^C^	0.056304
9	5.10 ± 0.05 ^Bb^	5.56 ± 0.02 ^Aab^	5.90 ± 0.02 ^Ba^	<0.000001
13	7.29 ± 0.06 ^Aa^	6.61 ± 0.11 ^Ab^	7.08 ± 0.04 ^Aab^	0.000004
*p* value time	0.000036	0.000454	0.000322	
*Enterobacteriaceae*				
0	2.03 ± 0.09 ^C^	1.71 ± 0.23 ^C^	2.02 ± 0.09 ^B^	0.069549
2	3.27 ± 0.69 ^Ba^	2.00 ± 0.15 ^Cb^	1.82 ± 0.10 ^Bb^	0.021316
6	4.41 ± 0.08 ^Ba^	3.43 ± 0.17 ^Bb^	3.10 ± 0.07 ^Ab^	0.000011
9	4.42 ± 0.06 ^Ba^	4.42 ± 0.09 ^Aa^	3.14 ± 0.08 ^Ab^	0.000011
13	5.54 ± 0.33 ^Aa^	4.25 ± 0.07 ^Ab^	3.23 ± 0.04 ^Ac^	0.002928
*p* value time	0.000772	0.003423	0.000062	
Psychrophiles				
0	2.89 ± 0.09 ^C^	2.73 ± 0.08 ^C^	2.86 ± 0.15 ^C^	0.207807
2	4.94 ± 0.32 ^B^	4.76 ± 0.15 ^B^	4.83 ± 0.16 ^B^	0.443364
6	6.58 ± 0.06 ^Aa^	5.52 ± 0.08 ^Ab^	5.04 ± 0.05 ^Bb^	<0.000001
9	7.08 ± 0.05 ^Aa^	5.99 ± 0.10 ^Ab^	5.33 ± 0.05 ^Bb^	<0.000001
13	7.66 ± 0.06 ^Aa^	6.74 ± 0.59 ^Ab^	6.73 ± 0.06 ^Ab^	0.000001
*p* value time	0.0000052	0.000001	<0.000001	

Data are presented as mean ± SD of 3 biological replicates in each condition and expressed as log CFU/g. MANOVA with categorical descriptors for the “time effect” and the “treatment effect” and Tukey’s post-hoc test were used as statistical analysis assuming *p* < 0.05 as significant. Within a specific group of microbes, the different lower case letters indicate statistical significance for the “treatment effect”, while different upper case letters indicate statistical significance for the “time effect”.

**Table 2:** Physico-chemical parameters of moisture content, pH and color (L*, a* and b* values) measured in the seabream fillets during refrigerated storage.

Storage Day	Control	Plasma-Air	Plasma-Argon	*p* Value Treatment
Moisture content (%)				
0	73.0 ± 1.0 ^aA^	73.6 ± 1.7 ^aA^	73.9 ± 0.6 ^aA^	0.234
2	73.3 ± 1.3 ^aA^	73.7 ± 0.4 ^aA^	74.2 ± 1.5 ^aA^	0.123
6	71.3 ± 1.2 ^aA^	72.2 ± 0.6 ^aA^	72.4 ± 0.9 ^aA^	0.342
9	72.4 ± 0.5 ^aA^	74.0 ± 0.3 ^aA^	72.1 ± 1.4 ^aA^	0.112
13	72.4 ± 2.1 ^aA^	74.1 ± 0.4 ^aA^	72.4 ± 2.1 ^aA^	0.254
*p* value time	0.114	0.210	0.301	
pH				
0	6.5 ± 0.1 ^aA^	6.5 ± 0.2 ^aA^	6.4 ± 0.2 ^aA^	0.213
2	6.4 ± 0.2 ^aA^	6.5 ± 0.1 ^aA^	6.5 ± 0.1 ^aA^	0.114
6	6.5 ± 0.1 ^aA^	6.5 ± 0.1 ^aA^	6.4 ± 0.2 ^aA^	0.126
9	6.5 ± 0.1 ^aA^	6.5 ± 0.2 ^aA^	6.4 ± 0.2 ^aA^	0.172
13	6.5 ± 0.2 ^aA^	6.5 ± 0.1 ^aA^	6.5 ± 0.1 ^aA^	0.138
*p* value time	0.154	0.134	0.08	
L*				
0	44.0 ± 1.4 ^bB^	47.7 ± 2.2 ^aA^	46.8 ± 1.5 ^aA^	<0.0001
2	43.9 ± 1.8 ^aB^	45.5 ± 2.9 ^aAB^	45.3 ± 1.6 ^aA^	0.158
6	44.5 ± 1.8 ^bB^	45.9 ± 1.7 ^abAB^	46.7 ± 2.1 ^aA^	0.025
9	44.6 ± 1.5 ^bB^	47.0 ± 2.1 ^aA^	46.7 ± 1.7 ^aA^	0.005
13	46.9 ± 1.6 ^aA^	44.4 ± 4.1 ^aB^	46.9 ± 1.6 ^aA^	0.110
*p* value time	0.004	0.002	0.008	
a*				
0	−4.0 ± 0.3 ^aA^	−3.8 ± 0.3 ^aA^	−3.9 ± 0.2 ^aA^	0.364
2	−3.4 ± 0.4 ^aA^	−3.2 ± 0.3 ^aA^	−3.4 ± 0.3 ^aA^	0.159
6	−3.3 ± 0.3 ^aA^	−3.3 ± 0.2 ^aA^	−3.5 ± 0.2 ^aA^	0.248
9	−3.3 ± 0.4 ^aA^	−3.3 ± 0.2 ^aA^	−3.4 ± 0.3 ^aA^	0.681
13	−2.8 ± 0.3 ^aB^	−2.7 ± 0.5 ^aB^	−2.8 ± 0.3 ^aB^	0.629
*p* value time	0.002	0.003	0.002	
b*				
0	−0.5 ± 1.7 ^aA^	−2.1 ± 1.0 ^bA^	−1.9 ± 0.6 ^bA^	0.008
2	−2.1 ± 1.2 ^aA^	−1.3 ± 0.8 ^aA^	−2.3 ± 1.1 ^aA^	0.074
6	−2.0 ± 1.1 ^aA^	−2.0 ± 0.9 ^aA^	−2.1 ± 0.9 ^aA^	0.984
9	−1.3 ± 1.0 ^aA^	−1.2 ± 1.0 ^aA^	−0.9 ± 1.3 ^aA^	0.603
13	−1.4 ± 1.1 ^aA^	−0.8 ± 0.9 ^aA^	−1.4 ± 1.1 ^aA^	0.193
*p* value time	0.007	0.121	0.007	

Data are mean ± SD of 3 biological replicates in each condition. MANOVA with categorical descriptors for the “time effect” and the “treatment effect” and Tukey’s post-hoc test were used as statistical analysis assuming *p* < 0.05 as significant. Within a specific group of quality parametr, the different lower case letters indicate statistical significance for the “treatment effect”, while different upper case letters indicate statistical significance for the “time effect”.

**Table 3:** Sensory score measured in the seabream fillets during refrigerated storage.

Storage Day	Control	Plasma-Air	Plasma-Argon
0	E	E	E
2	E	E	E
6	A	A	A
9	B	A	A
13	C	B	B

Excellent quality (E), good quality (A), fair quality (B), and unacceptable quality (C). Results are obtained by the evaluation of 3 fillets, by 6 panellists.

**Table 4:** TBARS and carbonyl content of not treated (control), Plasma-Air and Plasma-Argon treated fillets at 0 and 9 days of refrigerated storage.

Storage Day	Control	Plasma-Air	Plasma-Argon	*p* Value
TBARS (mg MDA/kg of sample)				
0	1.56 ± 0.52 ^a^	2.09 ± 0.14 ^a^	1.97 ± 0.69 ^a^	0.052
9	1.63 ± 0.19 ^b^	4.29 ± 0.34 ^a^	5.20 ± 1.06 ^a^	0.005
Carbonyl content (nmol/mg of protein)				
0	3.33 ± 0.81 ^a^	4.16 ± 1.36 ^a^	3.37 ± 0.82 ^a^	0.391
9	2.06 ± 0.62 ^a^	3.82 ± 0.54 ^b^	3.07 ± 1.28 ^b^	0.012

Data are mean ± SD of 3 biological replicates in each condition. Statistical analysis was by the one-way ANOVA using Tukeys’ as post-test and assuming *p* < 0.05 as significant. Different letters indicate statistical significance.

**Table 5:** Fatty acid composition (as FAME) of not treated (control), Plasma Air and Plasma Argon treated fillets at 0 and 9 days of storage.

	Control		Plasma-Air		Plasma-Argon		
	Storage Day						*p* Value
	0	9	0	9	0	9	
14:0	121.04 ± 14.01 ^a^	143.40 ± 49.58 ^a^	90.74 ± 8.48 ^a^	92.80 ± 43.22 ^a^	158.25 ± 115.17 ^a^	132.17 ± 79.40 ^a^	0.735
16:0	586.00 ± 61.00 ^a^	662.87 ± 202.81 ^a^	446.92 ± 32.18 ^a^	469.18 ± 191.84 ^a^	729.84 ± 478.17 ^a^	585.62 ± 300.03 ^a^	0.748
16:1 n-7	191.05 ± 18.12 ^a^	216.81 ± 71.16 ^a^	146.52 ± 27.14 ^a^	153.19 ± 73.00 ^a^	239.99 ± 168.23 ^a^	195.75 ± 111.69 ^a^	0.807
18:0	117.74 ± 11.77 ^a^	135.71 ± 34.80 ^a^	86.35 ± 3.17 ^a^	101.10 ± 33.98 ^a^	143.87 ± 90.73 ^a^	126.64 ± 61.09 ^a^	0.713
18:1 n-9	1046.92 ± 120.48 ^a^	1161.90 ± 373.91 ^a^	784.42 ± 109.14 ^a^	832.46 ± 353.32 ^a^	1358.82 ± 948.91 ^a^	1084.62 ± 614.09 ^a^	0.757
18:2 n-6	382.51 ± 41.61 ^a^	450.31 ± 144.80 ^a^	295.09 ± 32.43 ^a^	306.37 ± 110.39 ^a^	495.96 ± 334.64 ^a^	432.05 ± 235.71 ^a^	0.717
18:3 n-3	92.02 ± 10.09 ^a^	106.41 ± 36.52 ^a^	71.11 ± 9.93 ^a^	71.87 ± 25.31 ^a^	121.77 ± 84.53 ^a^	102.39 ± 57.51 ^a^	0.719
20:1	149.86 ± 16.72 ^a^	171.90 ± 53.77 ^a^	112.67 ± 11.28 ^a^	115.22 ± 53.49 ^a^	192.79 ± 136.74 ^a^	164.47 ± 96.64 ^a^	0.744
20:4	28.68 ± 2.55 ^a^	35.09 ± 8.37 ^a^	24.40 ± 2.89 ^a^	26.83 ± 6.55 ^a^	36.07 ± 19.62 ^a^	34.57 ± 12.88 ^a^	0.671
20:5 n-3	170.80 ± 13.96 ^a^	194.98 ± 60.42 ^a^	132.19 ± 13.20 ^a^	137.34 ± 25.54 ^a^	205.19 ± 117.02 ^a^	134.62 ± 1.30 ^a^	0.441
22:5 n-3	85.42 ± 8.12 ^a^	105.27 ± 36.94 ^a^	69.60 ± 8.88 ^a^	79.36 ± 26.90 ^a^	108.46 ± 66.37 ^a^	309.67 ± 347.85 ^a^	0.379
22:6 n-3	364.45 ± 33.17 ^a^	443.50 ± 90.23 ^a^	303.96 ± 26.21 ^a^	356.27 ± 83.16 ^a^	441.62 ± 232.82 ^a^	162.42 ± 229.70 ^a^	0.237
Total	3336.49 ± 348.00 ^a^	3828.13 ± 1163.13 ^a^	2563.98 ± 280.73 ^a^	2741.96 ± 1026.70 ^a^	4232.61 ± 2790.97 ^a^	3464.94 ± 1688.80 ^a^	0.739
SFA (%)	24.72 ± 0.18 ^a^	24.60 ± 0.03 ^a^	24.41 ± 1.00 ^a^	24.03 ± 0.81 ^a^	24.46 ± 0.61 ^a^	24.14 ±0.95 ^a^	0.822
MUFA (%)	41.57 ± 0.46 ^a^	40.39 ± 0.76 ^a^	40.61 ± 1.21 ^a^	39.65 ± 2.65 ^a^	40.59 ± 3.50 ^a^	40.75 ± 3.88 ^a^	0.962
PUFA (%)	33.71 ± 0.35 ^a^	35.01 ± 0.78 ^a^	34.98 ± 0.37 ^a^	36.32 ± 3.47 ^a^	34.94 ± 3.16 ^a^	35.11 ± 4.83 ^a^	0.923
n-3 PUFA (%)	21.38 ± 0.43 ^a^	22.35 ± 0.94 ^a^	22.52 ± 0.43 ^a^	24.11 ± 3.16 ^a^	22.54 ± 3.56 ^a^	21.81 ± 5.52 ^a^	0.911
n-6 PUFA (%)	12.32 ± 0.09 ^ab^	12.66 ± 0.16 ^ab^	12.46 ± 0.07 ^ab^	12.21 ± 0.31 ^b^	12.40 ± 0.50 ^ab^	13.30 ± 0.69 ^a^	0.044
Σ n-6/Σ n-3	0.57 ± 0.02 ^a^	0.57 ± 0.03 ^a^	0.55 ± 0.01 ^a^	0.51 ± 0.06 ^a^	0.56 ± 0.10 ^a^	0.64 ± 0.19 ^a^	0.6812

Single fatty acids are expressed as mg FAME/100 g sample; Σ SFA. MUFA. PUFA. PUFA n-3 and PUFA n-6 is expressed as mol/100 mol. Data are mean ± SD of 3 biological replicates in each condition. Statistical analysis was by one way ANOVA using Tukey’s as post-test. Different letters in the same row indicate significant differences (at least *p* < 0.05).

## Data Availability

The original contributions presented in the study are included in the article, further inquiries can be directed to the corresponding author.
